# Effect of bupivacaine intraperitoneal and intra-abdominal bicarbonate in reducing postoperative pain in laparoscopic cholecystectomy: a double-blind randomized clinical trial study

**DOI:** 10.1186/s13104-022-06083-3

**Published:** 2022-06-03

**Authors:** Nasim Nikoubakht, Seyed Hamid Reza Faiz, Seyed Hamzeh Mousavie, Amineh Shafeinia, Leila Borhani Zonoz

**Affiliations:** 1 Department of Anesthesiology and Pain Medicine, Rasool Akram Medical Complex, Iran University of Medical Sciences, Tehran, Iran; 2 Minimally Invasive Surgery Research Center, Department of Anesthesiology and Pain Medicine, School of Medicine, Iran University of Medical Sciences, Tehran, Iran; 3 Department of General Surgery, School of Medicine, Rasool Akram Medical Complex, Iran University of Medical Sciences, Tehran, Iran; 4 Department of Anesthesiology and Pain Medicine, Shahid Akbarabadi Hospital, Iran University of Medical Sciences, Tehran, Iran; 5 Nurse Anesthetist, Rasool Akram Medical Complex, Iran University of Medical Sciences, Tehran, Iran

**Keywords:** Pain, Bupivacaine, Bicarbonate, Cholecystectomy, Post-operative pain

## Abstract

**Objective:**

We aimed to compare the effect of bupivacaine intraperitoneal with intra-abdominal bicarbonate in reducing postoperative pain in laparoscopic cholecystectomy.

**Results:**

In this double-blind randomized clinical trial study, 58 patients underwent laparoscopic cholecystectomy referred to a hospital in Tehran, Iran (2019), were assigned into three groups: at the end of the surgery, spraying 50 cc of bupivacaine 0.2% through the laparoscopic port; or rinsing the abdomen with 5.7% bicarbonate dissolved in 1000 cc of normal saline; or abdominal lavage with normal saline. Pain of patients was evaluated according to visual analogue scale criteria and means Ramsay score in recovery times, 2, 8 and 24 h and post-operative analgesia satisfaction score at 2 and 24 h were also evaluated. The mean age of range was 44.26 ± 13.13 years, 44 female patients and 14 male patients. The mean Ramsay score in recovery, 2, 8 and 24 h postoperative times was not significantly different among the groups. Comparing post-operative analgesic satisfaction scores in recovery, 2 and 24 h revealed no significant difference among the groups. We found that use of bupivacaine intraperitoneal and intra-abdominal bicarbonate decreased pain after laparoscopic cholecystectomy but the decrease was more in bupivacaine group than bicarbonate group.

*Trial Registration:* Retrospectively registered, IRCT20180723040570N1; date of registration: 2019-06-24.

## Introduction

Pain is one of the complications of laparoscopic surgery. Untreated post-operative pain can interfere with sleep and physical activity and has negative effects on the patient's well-being [1]. Many studies have shown that the use of anti-inflammatory drugs declines complications after surgery but in most cases such measure is not enough [2]. Relieving postoperative pain, especially with certain types of analgesic agents, may reduce postoperative morbidity and mortality. It is also important to prevent adverse events such as myocardial infarction, cardiac arrhythmia, ileus, and poor wound healing and pulmonary complications [3–5].

Based on the patient’s preference and risk assessment there are several methods for controlling post-operative pain. These include systemic opioid and non-opioid analgesics, and regional, and neuroaxial analgesia. The use of local anaesthetics has been advocated as a method for reducing postoperative pain and drug use because it results in reducing drug-related side effects, improving patient recovery, and shortening of hospital stays [6, 7].

Epidural or intrathecal analgesia is considered as the gold standard for pain management in abdominal surgeries [8] but concerns about central block complications remain [9]. The local anaesthetic administration methods, however, are different. There is no strong evidence of the preference for a prescription method [10]. Recently, peripheral blocks and less invasive methods are more preferred for pain relief [11]. One such method is local anaesthetic intraperitoneal administration. It has shown good effects on reducing postoperative pain in laparoscopic cholecystectomy and gynaecological surgeries [8, 12–14]. Patients undergoing laparoscopic surgery experience postoperative pain especially in the abdomen, lower back, and shoulders, which requires adequate attention [15].

Most studies have discussed reducing postoperative pain for open surgeries [16] and the notion of most appropriate treatment for pain in patients undergoing laparoscopic surgery is still controversial. Recent studies have investigated the combination of local anaesthetic intraperitoneal with opioids [17]. Adjuvant drugs can reduce opioids usage and improve the quality of analgesia and this is particularly necessary for patients with a history of narcotic use as the latter show little response to the opioids [18].

The aim of this study was to compare the effects of bupivacaine intraperitoneal with intra-abdominal bicarbonate on reducing postoperative pain in laparoscopic cholecystectomy surgery.

## Main text

### Methods

The current study is a double-blind randomized clinical trial applied on 58 patients referred to the Rasool Akram Medical Complex in Tehran, Iran in 2019 for laparoscopic cholecystectomy. Inclusion criteria were age 17–60 years and ASA I and II. Exclusion criteria were patient dissatisfaction for participating in the study, opioid use within 24 h before the study, allergy to the drugs used in the study and alcohol use, chronic pain syndrome, neurological disease, steroid treatment, and the conversion of laparoscopic surgery to open surgery.

### Anaesthesia method and interventions

Protocol of anaesthesia induction was standard for all patients and included midazolam 25 µg/kg, fentanyl 2 µg/kg, as premedication, and propofol 2 mg/kg, atracurium 5 mg/kg and lidocaine 1.5 mg/kg. Anaesthesia maintenance was done by propofol 150–100 µg/kg with control of hemodynamic symptoms. For anesthesia depth, atracurium 0.2 mg/kg every 30 min was prescribed and morphine sulfate 0.1 mg/kg at the beginning of surgery during intra-abdominal injection of bupivacaine was used. Routine monitoring included non-invasive blood pressure (NIBP), Pulse oximetry (POM), EKG, and End-tidal CO_2_ (ET CO_2_). The ventilation condition was established so that the ET CO_2_ was maintained between 25 and 35. During the operation, the patient was placed in the trendelenburg reverse position and semi-sitting, and the intra-abdominal pressure was maintained between 15 and17 mmHg.

The required fluids were calculated according to the instructions for intraoperative fluid infusion. Patients were divided into three groups of normal saline, bicarbonate, and bupivacaine by permuted randomization. The patients and outcome assessors were unaware of the intervention type. The drug was administered during the surgery by a surgeon and the questionnaire was filled out by a nurse and she gave the researchers the questionnaire which had been assigned in three categories of A, B, C before the surgery. The evaluation after the surgery was performed by the researchers who were not aware of the categorization and they measured and screened the pain and satisfaction of the patients in recovery, 2, 8 and 24 h postoperative times. Also, if the patients had VAS higher than 3, pethidine was administered for their pain. All the patients received 2 g apotel.

The amount of narcotics and analgesics prescribed during the surgery was equal. At the end of the surgery, in the first group, 50 cc of bupivacaine 0.2% was sprayed by the surgeon through the laparoscopic port to wash the incisions and anastomosis, and after three minutes the abdomen was completely emptied of gas. In the second group, the abdomen was rinsed with 5.7% bicarbonate dissolved in 1000 cc of normal saline. While changing the patient’s position, the rinsing fluid was distributed throughout the abdomen. The patient was placed in the right and left lateral positions and trendelenburg position, and the supine position.

The third group underwent abdominal lavage with normal saline. Afterwards, the CO_2_ was drained from the peritoneal space. The anaesthesia was discontinued, and the patient was reversed with 40 µg/kg of neostigmine and 20 µg/kg of atropine, and then extubation was done. For all patients, an autofusor intravenous pain pump containing 2 g of apotel was administered within 24 h. The first request for analgesia was recorded by the nurse, and in case of pain in VAS more than 3, 0.5 mg/kg of pethidine was administrated. All surgeries were performed by one surgeon. Pain at recovery, 8, 12 and 24 h later were measured using VAS and patient’s satisfaction was measured by Ramsay score. Post-operation analgesic satisfaction score was also measured at the times for the three groups.

#### Statistical analysis

The sample size was calculated based on the study by Oza et al. [19] and using the following formula:

$$n\, = \frac{{z_{1}^{2} \, - \,\frac{\alpha }{2}\,\sigma^{2} }}{{d^{2} }}.$$

Descriptive results are presented as mean, standard deviation and frequency or percentage. Independent t-test was used to compare the two means. In case of abnormal data distribution, Mann–Whitney test was used. Also, Chi-square test was used to examine the differences of qualitative variables. Two-way Analysis of Variance Test (ANOVA) repeated measures analysis was used to evaluate the quantities between the groups and to examine the trend of intergroup pain. A P-value of less than 0.05 was considered statistically significant. All data were analyzed using SPSS software version 21.

## Results

**Fig. 1:**
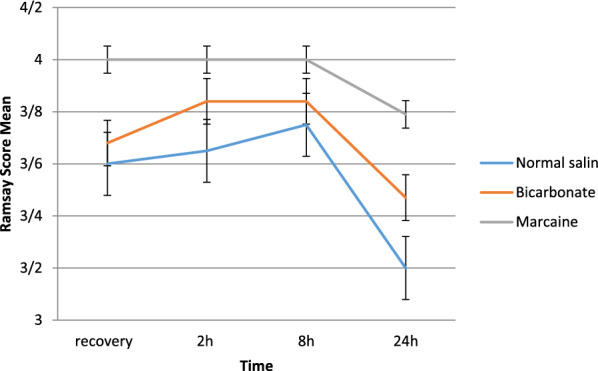
Trend, error bar, and mean Ramsay score of patients in recovery, 2, 8 and 24 h. The mean pain of patients was evaluated according to VAS criteria in recovery, 2, 8 and 24 h postoperative recovery times

This study was performed on 58 patients underwent laparoscopic cholecystectomy in 2019. The mean age of patients was 44.26 ± 13.13 years; 44 (75.9%) patients were female, and 14 (24.1%) patients were male. The basic characteristics of the patients in the three groups are shown in Table 1. The mean VAS (pain score) within the group in the measured times were evaluated using repeated measure. Results showed that the mean pain during the measured times had significant changes (P < 0.001). The results of intergroup effects showed that the differences between the two groups were statistically significant (P < 0.001) (Fig. 1).

**Table 1:** Basic characteristics of patients in the three groups of normal saline, bicarbonate and bupivacaine (marcaine)

Variable	Group			P value
	Normal saline (n=20)	Bicarbonate (n=19)	Marcaine (n=19)	
Sex				
Male (%)	5 (25.0)	5 (26.3)	4 (21.1)	1.000
Female (%)	15 (75.0)	14 (73.7)	15 (78.9)	
Underline Disease				
No (%)	16 (80.0)	13 (68.4)	16 (84.2)	0.536
Yes (%)	4 (20.0)	6 (31.6)	3 (15.8)	
Smoking				
No (%)	19 (95.0)	17 (89.5)	19 (100.0)	1.000
Yes (%)	1 (5.0)	2 (10.5)	0 (0.0)	
Age				
Mean ± SD	41.20±13.38	44.89±11.41	46.84±14.46	0.401
Body mass index				
Mean ± SD	24.49±7.18	25.65±4.01	24.56±2.89	0.730

The mean pain of the patient at all times was significantly different in the three groups (P < 0.05).

**Fig. 2:**
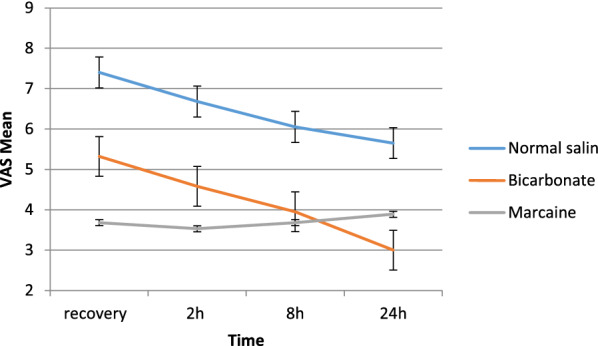
Trend and error bar, mean pain of the patients in recovery, 2, 8 and 24 h after surgery

The trends of intergroup pain in patients over time were evaluated, showing that the mean pain during the times had significant changes (P = 0.005). Results of intergroup effects showed that the differences between the two groups were statistically significant (P < 0.001) (Fig. 2). The mean of pethidine consumption showed that there was a significant difference in pethidine consumption (P = 0.029). Also, the mean consumption of apotel in the groups was not significantly different (P = 0.080). Mean Ramsay score was evaluated at recovery times, 2, 8 and 24 after surgery. Patients’ Ramsay scores were not significantly different in the three groups at all times (P > 0.05).

Post-operation analgesic satisfaction scores at 2 and 24 h was evaluated for the three groups. The results showed that the distribution for these variables was not different in the three groups (P > 0.05).

## Discussion

Uncontrolled pain after laparoscopic surgery is a clinical challenge that affects patient’s well-being significantly, leading to myocardial infarction, cardiac arrhythmia, ileus, and poor wound healing [1, 5]. Nowadays, there is an increasing tendency to alternative methods with the least side effects and less invasive for relieving pain such as intraperitoneal local anesthesia [11].

Bupivacaine as a long-acting local anesthetic reduces sodium permeability, increases the threshold of action, and prevents the conduction of nerve waves. This drug has a relatively slow onset (15 min) and long effect time (360–720 min) [20, 21]. On the other hand, the bicarbonate as an alkaline neutralizes the acidity caused by the gas, reducing the pain [22]. Based on our results, the bupivacaine group had the least pain and while the normal saline group as the control group had the highest mean VAS score. There was no significant difference between the groups regarding post-operation analgesic satisfaction score.

In a study by Stuhldreher et al. the effect of local anesthetic was evaluated on post-operative pain and the rate of drug use in patients underwent laparoscopic colorectal surgery. The first group received local anesthetic. The other group received both subcutaneous bupivacaine and intraperitoneal lidocaine. The study concluded that consumption of local anesthetics does not affect the amount of opioid required after the surgery [23].

Considering that the site of surgery in laparoscopic cholecystectomy is in the lower part of the chest, the patient's pain increases with each breath and the pain might be greater than that in colorectal surgery, resulting in different results in pain between the two studies. Compared to our study, there was no significant difference in the need for pain killer in any group, which supports the results of Stuhldreher’s study [23].

In another study, Joshi et al. managed pain after laparoscopic colorectal surgery by using ketorolac, methylprednisolone, intraperitoneal injection of ropivacaine, intravenous infusion of lidocaine, intrathecal morphine, and epidural anesthesia, which reduced the need for narcotics, and improved bowel function.

In conclusion, this study recommended surgical wound infiltration with local analgesia, systemic steroids, Non-steroidal anti-inflammatory drugs (NSAIDs), and specific Cyclooxygenase-2 (COX-2) inhibitors in combination with paracetamol and opioids [24]. Consistent with our results, the above study also confirms that the local anesthetic component among its interventions reduces pain after the surgery. They recommended use of adjuvant methods such as local anesthetics before the final suturing of the operation site in patients requiring optimal analgesia to reduce the dose of analgesics [24].

A study by Butala et al. [14] evaluated the effect of bupivacaine and intraperitoneal morphine on pain after laparoscopic gynecological surgery. Results showed that intraperitoneal administration of bupivacaine and morphine significantly reduces pain immediately after surgery. It also reduces the overall consumption of the life-saving drug 24 h without significantly increasing the side effects. Furthermore, the use of bupivacaine reduces postoperative pain; these results are similar to the current study, although in our study design, the need for apotel, and the dose of apotel in the treatment groups was not significantly different.

The results of the study showed that both methods, bupivacaine intraperitoneal and intra-abdominal bicarbonate, are effective in reducing opioid use and postoperative analgesia, leading to fewer side effects and better controlling the postoperative pain. In fact, the difference between the two methods is in the time of analgesia. In fact, the only difference between the two methods is in the time of analgesia.

### Limitation

One limitation of the current study is that none of the two persons were responsible for observing postoperative pain, that is, the surgeon who uses the drug and a nurse who filled questionnaire, had no role in analyzing and reviewing the results. This means that researchers could not check inter observer variability.

## Data Availability

The datasets used and/or analysed during the current study available from the corresponding author on reasonable request.

## Abbreviations

NSAIDs: Non-steroidal anti-inflammatory drugs
VAS: Visual analogue scale
NIBP: Non-invasive blood pressure
POM: Prescription only medicine
ET CO2: End tidal CO_2_
COX-2: Cyclooxygenase-2
PCA: Patient-controlled analgesia
ANOVA: Analysis of Variance Test
